# Prescribing patterns, indications and adverse events of ibuprofen in children: results from a national survey among Italian pediatricians

**DOI:** 10.1186/s13052-021-01047-y

**Published:** 2021-04-21

**Authors:** Massimo Martinelli, Lucia Quaglietta, Giuseppe Banderali, Pietro Ferrara, Claudio Romano, Annamaria Staiano

**Affiliations:** 1 Department of Translational Medical Science, Section of Pediatrics, University of Naples “Federico II”, Via S. Pansini, 5, 80131 Naples, Italy; 2 Santobono-Pausilipon Children’s Hospital, Naples, Italy; 3 Clinical Department of Pediatrics and Neonatology, San Paolo Hospital, ASST Santi Paolo e Carlo, University of Milan, Milan, Italy; 4 Institute of Pediatrics, Catholic University Medical School, Rome, Italy; 5 Service of Pediatrics, Campus Bio-Medico University, Rome, Italy; 6 Pediatric Gastroenterology and Cystic Fibrosis Unit, University of Messina, Messina, Italy

**Keywords:** Ibuprofen, NSAIDs, Pediatrics, Safety, Fever

## Abstract

**Background:**

Despite ibuprofen widely recognized safety profile, an increase of suspected adverse events has been reported in the last decade in parallel with its growing over-the-counter use. The aims of this study were to assess the therapeutic approach to the feverish child and to evaluate the main indications and the most frequent adverse events related to ibuprofen administration in children.

**Methods:**

A specific questionnaire-form regarding the management of ibuprofen therapy in children was distributed among a sample of pediatricians all over the Italian territory between September and October 2020. An electronic data collection through a specifically designed web-based platform was performed among the participating pediatricians.

**Results:**

One-hundred-eighty-one pediatricians completed the survey. In case of fever, 177 (98%) participants prescribe paracetamol, while only 4 (2%) preferred ibuprofen as first choice. One-hundred-twenty-eight pediatricians (71%) administer paracetamol alone, while 53 (29.2%) use the combined/alternating treatment with ibuprofen. Ibuprofen is mostly administered for musculoskeletal pain (30%), upper respiratory tract infection (20%), headache (15%) and post-surgical pain (9%). Sixty-three (35%) out of 181 participating pediatricians reported 191 adverse events during ibuprofen administration. The most common were gastrointestinal (GI), with GI bleeding being reported in 30/191 cases (15.7%), epigastric pain in 29/191 (15.1%), non-specified abdominal pain in 22/191 (11.1%) and nausea/vomiting in 21/191 (11%). Severe adverse events including kidney damage (3.1%), complicated infections (0.5%), pneumonia associated empyema (0.5%), soft tissue infection (0.5%) and disseminated intravascular coagulation (0.5%) were also reported. The adverse events led to a hospitalization in 12% of children. In 53/191 cases (28%) the adverse events were related to a wrong dosage or prolonged therapy or errors in frequency of administration.

**Conclusions:**

This survey demonstrate a sufficient awareness of Italian pediatricians regarding ibuprofen-prescribing patterns with the only possible concern related to the relatively high percentage of pediatricians performing a combining/alternating use of paracetamol and ibuprofen. The reported adverse events were mild in most of the cases and often related to errors in dosage, frequency and treatment duration, emphasizing the need for a major caution of both practitioners and patients in their use.

**Supplementary Information:**

The online version contains supplementary material available at 10.1186/s13052-021-01047-y.

## Introduction

Non-steroidal anti-inflammatory drugs (NSAIDs) include a group of worldwide used medications in both adults and children for their antipyretic and analgesic effects [1, 2]. Due to their anti-inflammatory properties NSAIDs are also administered during the course of pediatric inflammatory diseases such as juvenile idiopathic arthritis, Kawasaki disease and acute rheumatic fever [3, 4]. Fever is one of the most common symptoms in children [5], being responsible for about 20% of the consultations in primary care and emergency departments [6]. Being most of the time a physiological mechanism of defense, all the guidelines agree on antipyretics administration only with the purpose to control the child’s discomfort [2, 7, 8]. Ibuprofen, a non-selective cyclooxygenase (COX) inhibitor, is the currently recommended antipyretic to be used in pediatric age together with paracetamol, due to its tolerability/efficacy profile [9–11]. Current evidences suggest that there is no substantial difference between ibuprofen and paracetamol in term of safety and efficacy [12, 13], while combined or alternating use of paracetamol and ibuprofen is discouraged, considering risk and benefit [7, 8]. Despite ibuprofen widely recognized safety profile, an increase of suspected adverse ibuprofen related reactions has been reported in the last decade in parallel with its growing over-the-counter use [14, 15]. In most of the cases side effects involved the gastrointestinal (GI) system [16] and the kidneys [17], as a consequence of inappropriate administration or use. As a matter of fact, according to Italian post-marketing data, the proportion of packs of ibuprofen for pediatric use bought without a medical prescription increased from 28% in 2008 to 70% in 2015 [18]. In the light of emerging evidences, the aims of this study were to assess the therapeutic approach to the feverish child and to evaluate the main indications and the most frequent adverse events related to ibuprofen administration in children among a sample of Italian pediatricians.

## Methods

In July 2020 a panel of experts was selected within the Italian Society of Pediatrics. A face-to-face meeting resulted in organizing a specific questionnaire-form regarding the management of ibuprofen therapy in children to be distributed among a sample of Italian pediatricians all over the Italian territory. In details, the questionnaire-form included all the following items: type of pediatric working setting *(hospital pediatrician, general pediatrician or resident in Pediatrics)*; management of the feverish children, including which first drug was used and whether combined/alternating use of paracetamol and ibuprofen was performed; indications, dosage and duration of ibuprofen treatment; specific adverse events ibuprofen related and need for hospitalization; use of other NSAIDs and the specific indications. Overall, the questionnaire was structured with 19 items (Supplementary Table). The invitation to the survey was submitted twice by email, 15 days apart, between September 1st and September 22nd, 2020 to 8233 pediatricians belonging to the *Medikey database*. The *Medikey database* is an Italian national database listing most of the pediatrics specialists. An electronic data collection through a specifically designed web-based platform was performed among the participating pediatricians. The survey was completed on a voluntary basis and it was totally anonymous. To maximize the response rate a third reminder was sent in October 2020.

### Statistical analysis

All the data were collected in an excel database. Percentages and frequencies were summarized in order to report the proportion of different responses and were rounded to the nearest whole numbers. A descriptive analysis of the responses was performed using SPSS version 20 (SPSS Inc., Chicago, Illinois).

## Results

### Demographic data

Overall, 1467 out of 8233 (17.8%) pediatricians opened the email and 181/1467 (12.3%) completed the survey, of whom 124 were general pediatricians, 55 hospital pediatricians and 2 resident pediatricians.

### Management of the feverish child

**Fig. 1:**
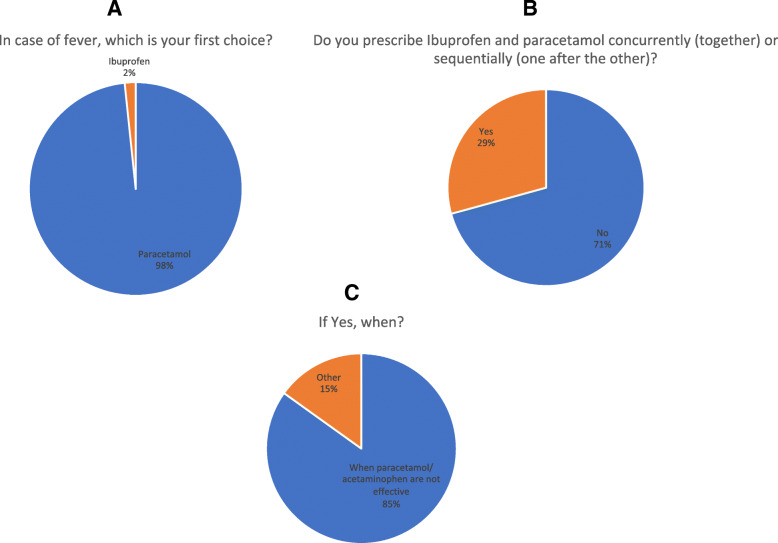
Management of feverish children among Italian Pediatricians. **a** Percentage of pediatricians prescribing paracetamol or ibuprofen as first-choice treatment for fever management; **b** Percentage of pediatricians using combined/alternating use of antipyretics; **c** Reasons for performing combined/alternating use of antipyretics in the feverish child

In case of fever, 177 (98%) participants prescribe paracetamol, while only 4/181 (2%) preferred ibuprofen as first choice *(*Fig. 1a). One-hundred-twenty-eight pediatricians (71%) administer paracetamol alone, while 53 (29.2%) use the combined/alternating treatment with ibuprofen (Fig. 1b). The reasons for combined/alternating use were paracetamol inadequate response (85%) or to have a prolonged effect (15%) during the night, in particular in patients with history of febrile seizures or inflammatory state (Fig. 1c).

### Ibuprofen: indications, rationale, age and formulations

**Fig. 2:**
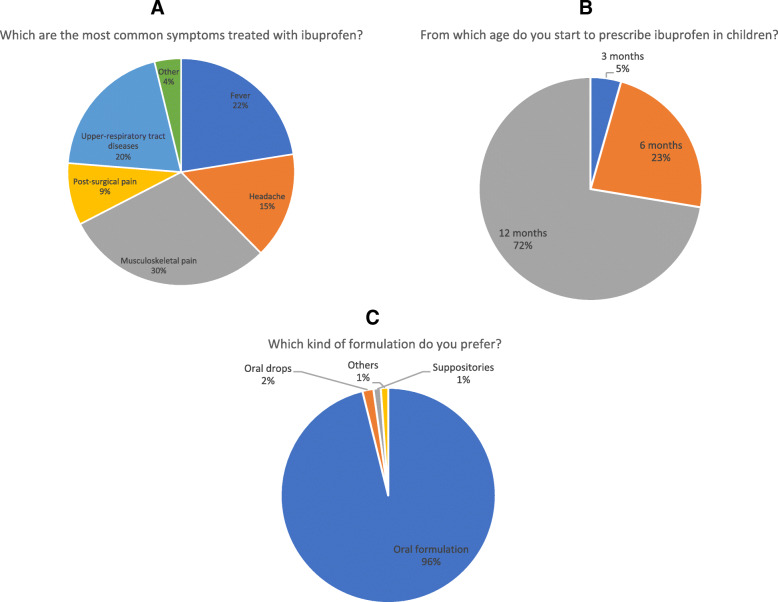
Ibuprofen: Indications, age and formulations. **a** Main indications for prescribing ibuprofen; **b** Starting age for Ibuprofen prescription; **d** Ibuprofen used formulations

Ibuprofen is mostly administered for inflammatory musculoskeletal pain (30%), upper respiratory tract infection (20%), headache (15%) and post-surgical pain (9%) (Fig. 2a). Also for these indications, 179 (99%) out of 181 pediatricians declared to use ibuprofen only if paracetamol was not effective. One-hundred-thirty (72%) participants administer ibuprofen in children of at least 1 year of age, 42 (23%) in children younger than 6 months of age and 9 (3%) in children starting from 3 months of age (Fig. 2b). The most used formulation resulted the oral suspension (98%), followed by oral drops (2%) (Fig. 2c).

### Ibuprofen treatment: dosage, duration, schedule and association with other NSAIDs

**Fig. 3:**
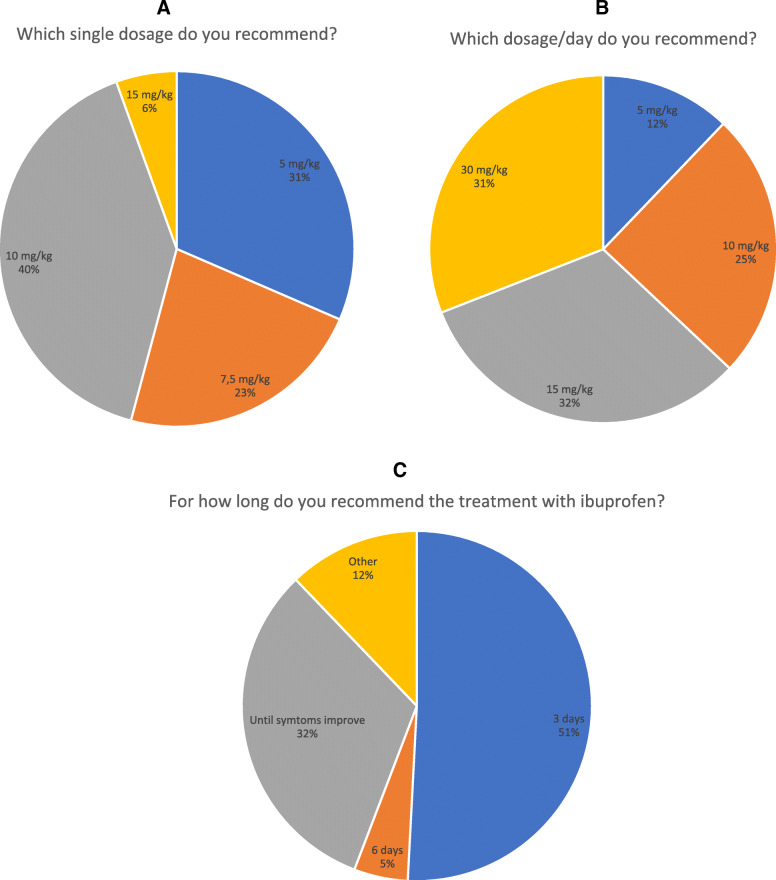
Ibuprofen treatment: doses, length, schedule and association with other NSAIDs; **a** ibuprofen dosage per dose; **b** ibuprofen dosage per day; **c** treatment duration

Each administration of ibuprofen mostly consisted in 10 mg/kg (41%), followed by 5 mg/kg (31%), 7.5 mg/kg (22%) and 15 mg/kg (7%). The time between administrations was 8 (69%), 6 (21%), 4 (6%) and 12 (4%) hours (69%) (Fig. 3a). Moreover, the suggested dosage per day was 15 mg/kg (35%), followed by 30 mg/kg (31%), 5 mg/kg (11%) and 10 mg/kg (23%) (Fig. 3b). The treatment is usually prolonged for 3 days in 54% of cases, even though 29% of pediatricians declared to continue the administration up to the disorder resolved (Fig. 3c). One-hundred-three (57%) participants routinely prescribe ibuprofen in association with other drugs, such as antibiotics (78%), paracetamol (20%), or other drugs (1%), such as anti-rheumatic drugs, immunosuppressants or biological drugs.

### Reported adverse events ibuprofen related

Sixty-three (35%) out of 181 participating pediatricians reported 191 adverse events during ibuprofen administration (Table 1). The most common adverse events were gastrointestinal, with GI bleeding being reported in 30/191 cases (15.7%), epigastric pain in 29/191 (15.1%), non-specified abdominal pain in 22/191 (11.1%) and nausea/vomiting in 21/191 (11%). Following GI side effects, the participating pediatricians described allergic reactions, including skin rash (21%), other hypersensitivity reactions (6.8%) and angioedema (2.1%) (Table 1). Severe adverse events including kidney damage (3.1%), complicated infections (0.5%), pneumonia associated empyema (0.5%), soft tissue infection during the course of chickenpox (0.5%) and disseminated intravascular coagulation (0.5%) were also reported (Table 1). Twenty-two out of 191 (11.5%) cases led to a hospitalization with the duration of 1 week in 13/22 (59%) and a couple of days in 9/22 (41%) of cases. In details, the longer hospitalizations were required for gastrointestinal bleeding (9/13, 69%), AKI (3/13, 23%) and disseminated intravascular coagulation (1/13, 8%). In 53/191 cases (28%) the adverse events were related to a wrong dosage or prolonged therapy or errors in frequency of administration. In 20/191 (10.4%) of the cases the children were contemporarily receiving a combined treatment with paracetamol, antibiotics or other NSAIDs. In case of adverse events, most of the pediatricians stopped the administration definitely (46%) or temporarily (43%), while 5% of the participants reduced the frequency of administration and 3% decreased the dosage.

**Table 1:** Reported adverse events following ibuprofen administration

Adverse event	N (%)
	(Total Number = 191)
Skin rash	40 (21)
Gastrointestinal bleeding	30 (15.7)
Epigastric pain	29 (15.1)
Abdominal pain	22 (11.5)
Nausea/Vomiting	21 (11)
Hypersensitivity	13 (6.8)
Acute kidney injury	6 (3.1)
Irritability	6 (3.1)
Diarrhea	5 (2.6)
Angioedema	4 (2.1)
Anemia	2 (1)
Liver damage	2 (1)
Dizziness	1 (0.5)
Insomnia	1 (0.5)
Bronchospasm	1 (0.5)
Non specified complicated infection	1 (0.5)
Pneumonia-associated empyema	1 (0.5)
Soft tissue infection during chickenpox	1 (0.5)
Leukopenia	1 (0.5)
Thrombocytopenia	1 (0.5)
Headache	1 (0.5)
Nasal bleeding	1 (0.5)
Disseminated intravascular coagulation	1 (0.5)

### Other NSAIDs

According to the survey results, 56 (31%) pediatricians routinely administer other NSAIDs including ketoprofen (45%), naproxen (27%), ketorolac (15%) or other (13%), such as flurbiprofen or clofoctol.

## Discussion

To the best of our knowledge this is the largest survey performed among Italian pediatricians to assess their therapeutic approach to the feverish child and their attitude regarding ibuprofen administration. Overall, the sample of pediatricians completing the survey showed a reasonable awareness of current recommendations on the management of the feverish children and tends to administer ibuprofen most often as a second choice both for fever and other indications. Although most of the guidelines discourage the combined/alternating regimen of paracetamol and ibuprofen, an alarming percentage of 29% of the participating pediatricians reported to routinely use this approach. As previously reported, ibuprofen seems to be well tolerated with a limited amount of adverse events, mostly mild, not leading to hospitalization and commonly related to errors in dosage, frequency of administration and treatment duration.

As reported above, fever is one of the most common reasons of primary care consultation and parental concerns [2, 18]. The underlying cause in childhood is generally benign, and fever has a beneficial effect in terms of fighting infection. However, fever can cause distress and discomfort in children, leading to a high degree of parental concern. So, for uncomplicated febrile children, the treatment is focused on comforting the children, also administering an antipyretic. Paracetamol and ibuprofen are the only antipyretics recommended for children [2, 7, 8]. In line with the current recommendations, the present survey showed that in case of fever, 98% of pediatricians tend to administer paracetamol, while only 2% of participants preferred ibuprofen as a first choice. Differently, despite the majority of the physicians completing the survey declared to avoid the combined/alternating regimen of paracetamol and ibuprofen, 53 (29.2%) of them still routinely administer this kind of regimen, especially in case of inadequate paracetamol response. The combined or alternating use of ibuprofen and paracetamol is still under debate [18, 19]. A Cochrane review including 6 studies with a total of 915 children found that the combined use of ibuprofen and paracetamol in febrile children results in a greater reduction in mean temperature after 1 and 4 h post administration compared to the use of a single antipyretic *(1 h mean difference: -0.27 °C; 95% CI-0.45 and − 0.08; 4-h mean difference: -0.70 °C; 95% CI-1.05 and-0.35)* [20]. In a randomized, comparative, trial including 99 feverish children aged from 6 months to 12 years, those receiving combination therapy with ibuprofen and paracetamol showed a significantly lower mean tympanic temperature, recorded 4 h after administration, when compared to children treated with paracetamol alone *(mean temperature reduction: 2.19 ± 0.83* vs *1, 48 ± 0.94; p < 0.05)* [21]. Although statistically significant, the difference in temperature decrease between was not considered clinically relevant [21]. On the other hand the safety of the association is under debate. As a matter of fact in 2014, Yue et al. analyzed the association of acute kidney injury (AKI) with ibuprofen, acetaminophen, and the combination of both drugs in children (0–12 years) by using the FDA Adverse Event Reporting System (AERS) database between January 2004 and June 2012 [22]. The combination of ibuprofen and acetaminophen was associated with AKI with an odds ratio of 4.01 (95% CI: 2.96–5.43) [22]. In summary, given the minimal benefits and considering the lack of safety studies, the most recent guidelines from national and international societies do not recommend the use of the alternating/combined regimen in children [7, 8]. Nevertheless, our survey shows that a non-irrelevant number of pediatricians do not fulfill this recommendation, highlighting the need for an increase of the current educational efforts.

When evaluating the answers to the survey, the general attitude regarding ibuprofen prescription was correct also for the other indications, the dosage and the length of treatment. As for the indications, ibuprofen was administered in musculoskeletal pain, upper respiratory tract infection, headache and post-surgical pain. Also in these cases pediatricians declare to mostly use it as a second-choice, after paracetamol failure. First-line treatment for mild-to-moderate inflammatory pain in childhood is either ibuprofen or paracetamol [23–25]. If pain relief is inadequate, second-line treatment is switching from one agent to the other, and third-line is treatment is to alternate between the two [23–25]. With regards to the dosage, in line with the current recommendations ibuprofen is mostly administered at 15 mg/kg/day, every 6–8 h for at least 3 days. When ibuprofen is administered at therapeutic doses in children the possible adverse events are, as for other NSAIDs related to inhibition of COX-1 and COX-2 and prostaglandin pathways [26].

In agreement with the previous literature, our data showed that 35% of Italian pediatricians observed specific adverse events during ibuprofen administration, with gastrointestinal symptoms being the most frequent. In 2016, Cardile et al. reported the results of a retrospective multicenter study conducted between January 2005 and January 2013 at 8 referral Italian pediatric gastroenterology centers aiming to characterize NSAIDs related GI bleeding [16]. Fifty-one children with GI bleeding were identified, with ibuprofen being the most frequently used NSAIDs. The authors concluded that GI bleeding after NSAIDs use is not uncommon, but often related to improper use, including self-prescriptions [16]. In line with these findings in 28% of the reported adverse events in our survey a wrong dosage, a prolonged therapy or errors in the frequency of administration were identified. These data once more highlight that in most of the cases the risk of NSAIDs adverse events can be prevented with their correct use. To this regard, it is worth underlining that the risk of wrong dosage may also be referred to the existence of different ibuprofen oral suspensions for children on the Italian market. The most commonly used formulations have 20 mg ibuprofen per ml, while a new oral suspension with double dosage (40 mg/ml) has recently been released. Despite its advantages in patients weighting> 30 Kg, its erroneous administration in younger children may increase the risk of overdosing.

Recently, a possible role of NSAIDs in worsening the clinical course of bacterial as well as viral infections has also been raised, especially for skin and soft tissue infections (SSTI) [27–29]. In our survey in 3/191 cases (1.6%) the participating pediatricians reported complicated infections, including empyema and STTI. Although very rare and still questioned, our findings confirm that Italian pediatricians are aware of the possibility of an increased risk of complicated infections following ibuprofen administration. However, further well-designed, trials are urgently needed to better define possible risk stratification and preventive strategies in pediatric patients.

Overall, the majority of the reported adverse events were mild, with only 12% of reporting need for hospitalization. In case of adverse events, most of participants just managed them either stopping the drug or decreasing the frequency of administration or the dosage. These data confirm the general safety of ibuprofen when it is used for proper indications and administered in a correct way.

It is acknowledged that the present study is not without limitations. The main drawbacks are related to the voluntary nature and to the low response rate of the survey, which may have selected the most virtuous pediatricians. In addition, the possibility of recall biases cannot be excluded. Additionally, we did not specifically evaluate the overall rate of self-prescriptions, which may certainly account for some of the described side effects. Otherwise, the main strength of the study lies in the high number of pediatricians, well distributed among the Italian territory, which give us a very precise picture of the ibuprofen administration in children.

## Conclusions

In conclusions, the results of our survey demonstrate a sufficient awareness of Italian pediatricians regarding ibuprofen-prescribing patterns both in the feverish child as well as in other indications. The only possible concern is related to the relatively high percentage of pediatricians performing a combining/alternating use of paracetamol and ibuprofen, which highlights the need for further educational efforts. The reported adverse events were mild in most of the cases and often related to errors in dosage, frequency and treatment duration, emphasizing once more the need for a major caution of both practitioners and patients in their use.

## Supplementary Information

**Additional file 1.**

## Data Availability

Not applicable.

## Abbreviations

AKI: Acute kidney injury
COX: Cyclooxygenase
GI: Gastrointestinal
NICE: National Institute for Health and Care Excellence
NSAIDs: Non-steroidal anti-inflammatory drugs
SSTI: Skin and soft tissue infections
