# Supplementary material for: Prescribing patterns, indications and adverse events of ibuprofen in children: results from a national survey among Italian pediatricians
Source: Ital J Pediatr. 2021 Apr 21;47:98. doi: 10.1186/s13052-021-01047-y (PMC8059227; doi:10.1186/s13052-021-01047-y)
Supplement: Supplementary file 1 — Additional file 1. [file 13052_2021_1047_MOESM1_ESM.doc]

| **Questions** |
| --- |
| 1. How many times per week do you prescribe ibuprofen? |
| - Never - Less than 5 times/week - 5-10 times/week - More than 10 times/week |
| 1. Which is/are the most common symptom(s) treated with ibuprofen? (multiple choices allowed) |
| - Fever - Musculoskeletal pain - Post-surgical pain (tonsillectomy or dental surgery) - Upper respiratory tract diseases (otitis media, rhinitis, tonsillopharyngitis) - Other |
| 1. In case of fever which is your first choice? |
| - Paracetamol/Acetaminophen - Ibuprofen |
| 1. Why do you prescribe ibuprofen to treat fever? |
| - This is my first choice - Paracetamol/Acetaminophen is not effective |
| 1. Do you prescribe Ibuprofen and paracetamol concurrently (together) or sequentially (one after the other)? |
| - No - Yes |
| If yes, when? |
| - When paracetamol/acetaminophen is not effective - Other |
| 1. From which age do you start to prescribe ibuprofen in children? |
| - 3 months - 6 months - 1 year |
| 1. Which kind of formulation do you prefer? |
| - Oral formulation - Oral drops - Suppositories - Other |
| 1. Which single dosage do you recommend? |
| - 5 mg/kg - 7.5 mg/kg - 10 mg/kg - 15 mg/kg |
| 1. Which dosage/day do you recommend? |
| - 5 mg/kg/day - 10 mg/kg/day - 15 mg/kg/day |
| 1. How long parents should wait to appreciate the efficacy? |
| - At least 4 hours - At least 6 hours - At least 8 hours - At least 12 hours |
| 1. For how long do you recommend the treatment with ibuprofen? |
| - 3 days - 6 days - Until symptoms improve - Other |
| 1. Do you prescribe ibuprofen in association with other drugs? |
| - No - Yes |
| If yes, please select the drugs below: |
| - Paracetamol/Acetaminophen - Other NSAIDs - Antibiotics - Other |
| 1. Did you ever observe any ibuprofen-related adverse event? |
| - No - Yes |
| If yes, please select the adverse event  If no, please select which one do you consider ibuprofen-related adverse event |
| - Epigastralgia - Urticaria - Abdominal pain - Skin Rash - Hypersensitivity - Hematemesis - Vomit - Gastrointestinal bleeding - Kidney damage - Nausea - Irritability - Diarrhoea - Dyspepsia - Laryngeal oedema - Anaemia - Rectorrhagia - Hematemesis and rectorrhagia - Angioedema - Dizziness - Insomnia - Bronchospasm - Complicate infections - Pneumonia-associated empyema - Soft tissue infection in patients with varicella zoster - Liver failure - Leucopoenia - Thrombocytopenia - Headache - Others |
| 1. During the treatment with ibuprofen, did you ever experience: |
| - Complicated infections - Soft tissue infection in patients with varicella zoster - Pneumonia-associated empyema - Other |
| 1. Did any of those adverse events require hospitalization? |
| - Yes - No |
| If yes, for how long? |
| - 2 days - 1 week - Other |
| 1. Did the patients present any concomitant conditions? |
| - Yes - No |
| If yes, select the condition below: |
| - Exanthematous disease - Infections - History of other diseases (please describe) |
| 1. In your opinion, which ones are the reasons for adverse events in ibuprofen? |
| - Wrong dosage - Wrong schedule of administration - Combined therapy (please specify which drug) - Long-lasting therapy - Other |
| 1. Which is your strategy in case of ibuprofen-related adverse events? |
| - Stopping the treatment (temporarily) - Stopping the treatment (definitely) - Reducing dosage - Reducing the frequency of administration - Other (please specify) |
| 1. Do you prescribe any other NSAIDs to children (other than ibuprofen)? |
| - No - Yes |
| If yes, please specify: |
| - Ketoprofen - Naproxen - Ketorolac - Other (please specify) |
